# Difference in aqueous concentration and vitreous mass of cytokines in high myopias with and without choroidal neovascularization

**DOI:** 10.3389/fmed.2022.1029425

**Published:** 2022-11-10

**Authors:** Shian Zhang, Jianbo Mao, Nuo Chen, Yuyan Fang, Yijing Chen, Zicheng Zheng, Yiqi Chen, Lijun Shen

**Affiliations:** ^1^Department of Ophthalmology, Center for Rehabilitation Medicine, People's Hospital of Hangzhou Medical College, Zhejiang Provincial People's Hospital, Hangzhou, China; ^2^Eye Hospital and School of Ophthalmology and Optometry, Wenzhou Medical University, Wenzhou, China; ^3^Hwa Mei Hospital, University of Chinese Academy of Sciences, Beijing, China

**Keywords:** cytokine, VEGF, high myopia, choroidal neovascularization, axial length

## Abstract

**Purpose:**

To investigate and compare the aqueous humor (AH) concentration and vitreous mass of cytokines in high myopias (HM) with and without myopic choroidal neovascularization (mCNV). And the correlations between cytokines and the size of CNVs on optical coherence tomography angiography (OCTA) images were also be analyzed.

**Methods:**

This observational study included 56 highly myopic eyes with and without CNV and 57 control eyes with cataracts. AH samples were obtained prior to the intravitreal injection of anti-VEGF and cataract surgery. This study measured multiple inflammatory cytokines including VEGF, interleukin (IL)-6, IL-8, IL-10, interferon-inducible protein-10 (IP-10), and monocyte chemotactic protein-1 (MCP-1) by multiplex bead assay. AH cytokine level, axial length, and vitreous volume were used to calculate the vitreous mass of cytokines.

**Results:**

The vitreous mass of VEGF in eyes with mCNV was significantly higher than that in control group. However, the difference in AH concentration of VEGF between high myopias with mCNV was not observed. Inflammatory cytokines were upregulated (IL-6, IL-10, and MCP-1) in highly myopic eyes both with and without mCNV (all P<0.05). There was also a significant difference in the vitreous mass of IL-8 and IP-10 among all three groups (P<0.05).

**Conclusion:**

We confirmed the secretion of VEGF increased in eyes with mCNV from a new perspective. The development of both HM and mCNV were related to inflammatory cytokines and the upregulation of inflammatory cytokines may precede upregulation of VEGF. The vitreous mass might be tried as a more reliable potential biomarker in eyes with longer axial length.

## Introduction

Myopia is a leading cause of visual impairment worldwide with a high prevalence especially in young adults in East Asia, ranging from 80 to 90% (1, 2). Approximately one-fifth of the myopic population has high myopia (≥-6 diopters) which can be associated with multiple kinds of retinochoroidal lesions such as myopic choroidal neovascularization (mCNV) (1). If left untreated in a period as short as 5 years, CNV can cause scarring with expanding macular atrophy leading to irreversible visual loss (3, 4).

Vascular endothelial growth factor (VEGF) is implicated in the pathogenesis of CNV, making intravitreal injection of anti-VEGF the first-line treatment (5). However, 15% of patients in our earlier study who had VEGF levels within the normal range were resistant to anti-VEGF medication (6). Aqueous humor (AH) concentration of VEGF was investigated as a therapeutic biomarker of anti-VEGF treatment efficacy (7) as it was strongly correlated with the concentration of VEGF in vitreous humor (8) which reflected the retinal microenvironment directly in normal axial eyes. Anti-VEGF therapy is effective for most CNV diseases, including mCNV. However, it was contradictory that the AH concentration of VEGF in mCNV eyes was generally decreased compared to the control subjects in earlier investigations (9, 10). Recently, Wei et al. discovered that the concentration of VEGF was much higher in vitreous humor samples from high myopia (HM) eyes which confirmed the secretion of VEGF increased, supporting the hypothesis about the lower AH concentration of VEGF that the longer axial length and larger intraocular volume diluted the VEGF concentration in AH. However, because it is obviously not justified to collect the vitreous samples from each high myopia patient, AH samples remain the current first choice for assessing intraocular VEGF levels (8). Since the traditional AH concentration may not be suitable for the assessment of overall intraocular VEGF levels in long axial eyes, a new metrics need to be explored.

The inflammatory internal microenvironment in HM eyes also plays an important role in the progression of CNV and myopia. AH and vitreous concentration of inflammatory cytokines, such as interleukin-8(IL-8), and monocyte chemotactic protein-1 (MCP-1), and interferon-inducible protein-10(IP-10), were significantly increased in CNV diseases (11–13). And these cytokines have been demonstrated to be mediators of inflammation and angiogenesis in various ocular and systemic diseases (12), which also induces VEGF upregulation while participating in the inflammatory response (6). Furthermore, pro-fibrotic cytokines which usually function additively or synergistically with inflammatory cytokines, were found to be significantly higher in HM eyes without CNV (8). However, the complex cytokine network in the eye needs to be further investigated, and the dilution effect in HM eyes poses a difficulty for research.

This study aimed to compare the aqueous concentrations of VEGF and inflammatory cytokines in HM eyes (with and without CNV) and relatively normal axial eyes. To minimize the interference of dilution effects, we tried to calculate and compare the vitreous mass of cytokines based on the estimated vitreous volume size according to the axial length. The correlation between the cytokine and the size of CNV area was also analyzed.

## Methods

This observational study was conducted between Nov 2021 and May 2022 and included 113 eyes for the treatment of active mCNV or only for cataract surgery. The study was approved by the Ethics Committee and the procedures followed the tenets of the Declaration of Helsinki (IRB number KY2022022). To participate in the trial, every patient signed a written informed permission form.

The inclusion criteria were as follows: (1) Over the age of 18, (2) No previous intraocular surgery or intravitreal injections, (3) spherical equivalent < −6.0 D, and axial length >26 mm. The exclusion criteria were were as follows: (1) any other retinopathy, such as diabetic retinopathy, retinal vascular occlusion, or retinal detachment; (2) uveitis; (3) glaucoma and/or iris neovascularization; and (4) any prior treatment for CNV, such as laser therapy within the preceding 6 months or PDT.

Based on the ocular status with or without active CNV, patients were divided into mCNV group and high myopias (HM) without CNV group (hereafter HM group). The presence of CNVs had to be confirmed by both fluorescein angiography (FA) and spectral domain optical coherence tomography (SD-OCT), consisting of late leakage of the hyperfluorescent neovascular network and the presence of sub/or intraretinal fluid and/or subretinal hyperreflective exudation, respectively. The control group, which included 57 eyes that underwent cataract surgery but did not have any additional ocular or immune-mediated diseases, served as comparison.

Before receiving an anti-VEGF injection or having cataract surgery, all patients underwent a thorough ophthalmological examination that included a measurement of their best-corrected visual acuity (BCVA), which was expressed as the log of the minimum angle of resolution (logMAR), intraocular pressure, slit-lamp biomicroscopy, and a dilated fundus examination. Axial length was measured using the IOL-Master 700 (Carl Zeiss Meditec, Jena, Germany). Zhou et al. analyzed the topography of human eyes with pathological myopia through volume rendering images by high-resolution 3D- magnetic resonance imaging (MRI) and established a model to estimate the vitreous volume (14). The estimation formula of vitreous volume is as follows:

- HM eyes: Vitreous volume (mm^3^) = 546.27 × axial length (mm)-6977.12
- Controls: Vitreous volume (mm^3^) = 458.35 × axial length (mm)-6331.14.

The Heidelberg Spectralis (Heidelberg Engineering, Heidelberg, Germany) performed SD-OCT imaging on all patients 1 to 3 days before the procedure. We defined the central macular thickness as the average retinal thickness within the 1-mm diameter central field of the Early Treatment Diabetic Retinopathy Study. The optical coherence tomography angiography (OCTA) images were produced using a split-spectrum amplitude decorrelation angiography (SD-OCT) equipment (RTVue-XR Avanti; Optovue, Inc, Fremont, CA).Then the size of CNVs was marked with the manual selection tools and calculated based on the built-in software (11). Images with poor quality (quality <7) were excluded from the analysis.

Before intravitreal injection or cataract surgery, AH samples were taken. Approximately 0.05–0.1 mL of AH was withdrawn using a 30-gauge insulin syringe *via* limbal paracentesis. Each sample was immediately transferred to a sterile plastic tube and kept at −84°C until assayed. VEGF, interleukin (IL)-6, IL-8, IL-10, interferon-inducible protein-10 (IP-10), and monocyte chemotactic protein-1 (MCP-1) were measured in undiluted AH samples by Luminex200 (BIO-RAD, Hercules, CA, USA). Each cytokine concentration (pg/mL) was calculated based on the standard curve provided by the kit. All procedures were carried out in a dark environment with room temperature illumination to prevent degradation brought on by light (6, 11). Previous studies found that the majority of the proteins presented in both aqueous and vitreous fluid were in similar quantities (15) and cytokines such as VEGF demonstrated a strong correlation in vitreous and aqueous of patients (16). We assume tentatively that the concentration of cytokines in the AH is approximately equal to the concentration in the anterior vitreous. Thus, the AH concentration is used to estimate the mass in the vitreous, and the following formula was yielded:

Vitreous mass (pg)= Aqueous Humor Concentrations (pg/mL) × Vitreous Volume (mm^3^) × 10^−3^(mL/mm^3^).

SPSS for Windows was used to execute each and every statistical analysis (version 26.0, SPSS Inc., Chicago, IL, USA). Means and standard deviations were used to express continuous variables. The Shapiro-Wilk test was used to determine whether the distribution of the cytokine data was normal. Non-parametric analysis of variance was used to compare groups using either the Mann-Whitney U test or the Kruskal-Wallis test for data that were not normally distributed. Significant values for the Kruskal-Wallis test were modified using the Bonferroni correction for multiple tests on continuous variables. The Spearman rank correlation analysis was used to determine the correlations between the variables. Statistics were judged significant at P <0.05.

## Results

In this observational study, we included 113 eyes, including 30 highly myopic eyes (9 males and 21 females) with active mCNV, 26 highly myopic eyes (5 males and 21 females) without CNV and 57 control eyes (19 males and 38 females). The mean age was 57.6 ± 16.0 years in the mCNV group, 63.0 ± 8.3 years in the HM group and 69.5 ± 9.2 years in the control group (Table 1). The axial length and vitreous volume of eyes with HM (with or without CNV) were significantly higher than those of the control group (both *P* < 0.001). Although the control group was older than the other groups, there were no correlations between age and cytokines (*P* > 0.05 for all, Table 2).

**Table 1:** Differences in age, sex, axial length, and vitreous volume in the three groups.

	**Control group**	**HM**	**mCNV**	P*	**P^†^**	**P^‡^**
Age	69.5 ± 9.2	63.0 ± 8.3	57.6 ± 16.0	**0.027**	**0.001**	1.000
Gender (M:F)	19:38	5:21	9:21	0.296	0.813	0.537
Axial length (mm)	23.22 ± 0.91	29.82 ± 1.69	28.51 ± 2.20	**<0.001**	**<0.001**	0.647
Vitreous volume (mm3)	4310.46 ± 415.10	9311.08 ± 923.89	8597.95 ± 1199.80	**<0.001**	**<0.001**	0.655

HM, High myopia without choroidal neovascularization group; mCNV, myopic choroidal neovascularization group. P*:control vs. HM, P^†^:control vs. mCNV, P^‡^:HM vs. mCNV. Bold values to highlight significant difference (P < 0.05).

**Table 2:** Correlation between age and cytokines in the control group.

**Correlation**	**VEGF**	**IL-6**	**IL-8**	**IL-10**	**IP-10**	**MCP-1**
P	0.872	0.326	0.334	0.795	0.640	0.082

VEGF, vascular endothelial growth factor; IL-6, interleukin 6; IL-8, interleukin 8; IL-10, interleukin 10; IP-10, interferon-inducible protein 10; MCP-1, monocyte chemotactic protein 1. P-values calculated by Spearman rank correlation analysis.

### AH concentration of cytokines

The differences in AH concentration of the cytokines in the three groups were shown in Table 3. The AH concentration of VEGF in the control group and mCNV group were both higher than that in the HM group (*P* < 0.001 and = 0.008, respectively). However, there was no difference between the control group and the mCNV group (*P* = 1.000).

**Table 3:** Aqueous humor concentrations (pg/mL) of cytokines in the three groups.

**(pg/ml)**	**Control group**	**HM**	**mCNV**	P*	**P^†^**	**P^‡^**
VEGF	37.18 ± 20.09	21.19 ± 13.14	33.74 ± 15.38	**<0.001**	1	**0.008**
IL-6	8.17 ± 8.70	22.03 ± 21.99	11.87 ± 15.80	**0.002**	0.825	0.067
IL-8	11.69 ± 8.73	10.19 ± 12.28	19.00 ± 12.95	0.36	**0.004**	**<0.001**
IL-10	0.81 ± 0.26	0.84 ± 0.20	0.92 ± 0.18	0.085	0.085	0.085
IP-10	239.01 ± 199.56	243.95 ± 123.03	619.76 ± 577.38	0.952	**<0.001**	**<0.001**
MCP-1	471.80 ± 229.21	570.80 ± 158.50	766.33 ± 496.15	**0.037**	**<0.001**	0.582

HM, High myopia without choroidal neovascularization group; mCNV, myopic choroidal neovascularization group; VEGF, vascular endothelial growth factor; IL-6, interleukin 6; IL-8, interleukin 8; IL-10, interleukin 10; IP-10, interferon-inducible protein 10; MCP-1, monocyte chemotactic protein 1. P*:control vs. HM, P^†^:control vs. mCNV, P^‡^:HM vs. mCNV. Bold values to highlight significant difference (P < 0.05).

The AH concentration of IL-8 and IP-10 in the mCNV group was higher than those in the control group (*P* = 0.004 and = 0.001, respectively) and the HM group (both *P* < 0.001). However, there was no difference between the control group and the HM group (*P* = 0.360 and 0.952, respectively).

The AH concentration of MCP-1 in the mCNV group and HM group was higher than that in the control group (*P* < 0.001 and = 0.037). However, there was no difference between the mCNV group and HM group (*P* = 0.582).

### Vitreous mass of cytokines

The differences in vitreous mass of the cytokines in the three groups were shown in Table 4. The vitreous mass of VEGF in the mCNV group was significantly higher than that in the control group and HM group (*P* < 0.001 and = 0.014, respectively). However, there was no difference between the control group and HM group (*P* = 0.771).

**Table 4:** Vitreous mass (pg) of cytokines in the three groups.

	**Control group**	**HM**	**mCNV**	P*	**P^†^**	**P^‡^**
VEGF	160.69 ± 88.54	197.12 ± 125.40	286.65 ± 126.91	0.764	**<0.001**	**0.014**
IL-6	34.68 ± 36.13	205.57 ± 209.42	106.13 ± 151.11	**<0.001**	**0.001**	0.078
IL-8	49.79 ± 35.99	93.81 ± 115.32	165.99 ± 118.54	**0.014**	**<0.001**	**0.013**
IL-10	3.52 ± 1.25	7.84 ± 2.02	7.9 ± 1.91	**<0.001**	**<0.001**	1
IP-10	1031.34 ± 882.17	2235.28 ± 998.72	5399.93 ± 5278.21	**<0.001**	**<0.001**	**0.017**
MCP-1	2024.6 ± 964.49	5326.76 ± 1582.40	6650.08 ± 4539.49	**<0.001**	**<0.001**	1

HM, High myopia without choroidal neovascularization group; mCNV, myopic choroidal neovascularization group; VEGF, vascular endothelial growth factor; IL-6, interleukin 6; IL-8, interleukin 8; IL-10, interleukin 10; IP-10, interferon-inducible protein 10; MCP-1, monocyte chemotactic protein 1. P*:control vs. HM, P^†^:control vs. mCNV, P^‡^:HM vs. mCNV. Bold values to highlight significant difference (P < 0.05).

The vitreous mass of IL-6, IL-8, IL-10, IP-10, and MCP-1 in the HM group and mCNV group were both higher than those in the control group (all *P* < 0.05). However, for all these cytokines except IL-8 and IP-10 (*P* = 0.013 and 0.017, respectively), there was no difference between the HM group and mCNV group (all *P* > 0.05).

### Correlation between cytokines and CMT size of CNV

In the mCNV group, the AH concentration and vitreous mass of cytokines had no correlation with CMT or size of CNV (Table 5).

**Table 5:** Correlations between aqueous humor concentration / vitreous mass of cytokines and CMT/size of CNV in the mCNV group.

**Concentration**	**CMT**	**Size of CNV**	**Mass**	**CMT**	**Size of CNV**
VEGF	−0.044	0.079	VEGF	0.032	0.156
P	0.817	0.679	P	0.867	0.411
IL-6	0.21	0.306	IL-6	0.24	0.334
P	0.266	0.1	P	0.201	0.071
IL-8	−0.119	0.103	IL-8	−0.08	0.178
P	0.532	0.588	P	0.673	0.347
IL-10	0.09	0.131	IL-10	0.181	0.31
P	0.636	0.49	P	0.338	0.095
IP-10	−0.231	0.019	IP-10	−0.176	0.102
P	0.22	0.923	P	0.352	0.591
MCP-1	−0.113	−0.12	MCP-1	0.009	−0.015
P	0.552	0.528	P	0.962	0.937

HM, High myopia without choroidal neovascularization group; mCNV, myopic choroidal neovascularization group; CMT, central macular thickness; VEGF, vascular endothelial growth factor; IL-6, interleukin 6; IL-8, interleukin 8; IL-10, interleukin 10; IP-10, interferon-inducible protein 10; MCP-1, monocyte chemotactic protein 1.

## Discussion

The main findings in the present study were as follows: (1) The mass of VEGF in eyes with mCNV was significantly higher than that in the normal eyes, while there was no significant difference in AH concentration of VEGF between the two groups. It is speculated that the occurrence and development of CNV were mainly mass-dependent rather than concentration-dependent; (2) Some inflammatory cytokines (IL-6, IL-10, and MCP-1) in highly myopic eyes have been upregulated regardless of the presence or absence of CNV. (3) The mass of IL-8 and IP-10 gradually increased with the severity of HM.

VEGF is an angiogenic and vasopermeability factor. Progressive distension of the posterior pole stretches the RPE cells and changes the choroidal hemodynamic, which leads to a decrease in choroidal perfusion. This further induces choroidal ischemia that upregulates the expression and secretion of VEGF, resulting in development of mCNV (17). However, AH concentration of VEGF in mCNV is still controversial. Several studies demonstrated that the VEGF level in eyes with mCNV is generally lower than that in control subjects (myopic patients without CNV or patients without myopia) (9, 18, 19), which is inconsistent with another report in which the VEGF level was shown to be increased (20).

An explanation is that VEGF concentration is gradually diluted with diffusion from the vitreoretinal interface to the AH of the anterior chamber, and the dilution effect is more obvious in the eyes with high myopia due to the longer axial length and larger intraocular volume. For the HM without CNV and mCNV groups, since the two are similar in ocular size and the dilution effect could be almost ignored, and active neovascularization were presented in the latter group, it could be explained why the VEGF level was increased in eyes with mCNV. However, when compared to controls, it could present two completely opposite findings, increased or decreased VEGF, since the secretion of VEGF and axial length (or intraocular volume) both increased (21). Our results were similar to the previous studies showing that the VEGF concentrations were significantly lower in HM eyes with or without CNV when compared with controls and that the VEGF concentration in mCNV was significantly higher than in HM without CNV (9, 18, 19), but we did not observe the difference between the control group and mCNV group.

To eliminate the influence of the volume, we used the axial length to estimate the vitreous volume and multiplied it by the concentration of VEGF. Then we compared the difference in mass among different groups instead of concentration. In our results, the vitreous mass of VEGF in the mCNV group was higher than that in the HM group and control group. It confirms our conjecture that the concentration could not accurately reflect the actual intraocular secretion and upregulation of VEGF due to the large individual variation in axial length, which differs from the previous studies on cytokines in other retinopathies that included only eyes with normal axial length. This finding is also consistent with our cognition that VEGF is upregulated in the mCNV and explains the occurrence of CNV in HM patients but not in controls under conditions of similar AH concentrations of VEGF. Recently, Wei et al. found that the concentration of VEGF was significantly increased in the vitreous humor samples from HM (22), which contradicts the view that retinal thinning might cause relatively increased choroidal perfusion and decreased retinal hypoxia resulting in decreased VEGF production (9), However, in our results, there is no significant difference in the vitreous mass of VEGF between HM group and the controls. This discrepancy might be due to the different inclusion criteria since Wei et al. included HM patients with rhegmatogenous retinal detachment, myopic retinoschisis, idiopathic epiretinal membrane, or macular hole (22).

Yuan et al. reported the possible connection between highly myopic eyes and low-grade or subclinical inflammation (10). For the mass of IL-6, IL-10, and MCP-1, these inflammatory cytokines had significantly increased before mCNV had occurred in highly myopic eyes. Although the mass relationships between different groups in our results were obtained through indirect calculations, they are very similar to the relationship acquired through direct measurement from vitreous samples by Wei et al. (22) i.e., the vitreous concentrations of IL-6, IP-10 and MCP-1 were significantly elevated in the HM patients. IL-6 is a pro-inflammatory cytokine involved in angiogenesis by VEGF induction and correlated with AH concentration of VEGF (23). Zhao et al. (24) demonstrated that the upregulation of MCP-1 contribute to axial length elongation and myopia development. VEGF-related pathways drive MCP-1-induced *in vivo* angiogenesis, and a positive regulatory feedback loop between VEGF and MCP-1 production may intensify inflammatory reactions and angiogenesis (6). Since IL-10 is an immunomodulatory cytokine (25), it is reasonable to assume that the IL-10 secretion was a compensatory response before the appearance of CNV and that the sustained decompensation of IL-10 may lead to the CNV formation.

The AH concentrations of IL-8 and IP-10 were increased in the mCNV group compared to controls and the HM group in our result, and similar findings were also obtained by Yamamoto et al. (18). In addition, we found the mass of IL-8 and IP-10 gradually increased with the severity of high myopia, suggesting that the associated low-grade retinal inflammation may occur prior to the appearance of neovascularization. IL-8 stimulates the expression of VEGF and the activation of VEGF receptors in vascular endothelial cells, and was also found in elevated concentrations in CNV (18, 26). IP-10 have also been demonstrated to be mediators of inflammation and angiogenesis in various ocular diseases (12). IL-8 and IP-10 may be therapeutic targets or markers of disease progression.

In the mCNV group, the AH concentration and vitreous mass of cytokines had no correlation with CMT or size of CNV. The possible explanations are as follows: In other CNV diseases, such as AMD, type 1 CNV did not affect visual activity in the early stages and patients seek medical attention later, when the size of CNV was large and VEGF level were significantly elevated (11). In contrast, the majority of mCNV are type 2 CNV, which rapidly affected vision as soon as it appeared. The CNV area was still minor when the majority of patients received timely therapy. In our results, the majority of mCNV eyes had modest CNVs, hence there was no significant relationship between cytokines and size of CNV. The correlation with CMT was similar.

There are some limitations of this study. Firstly, the difference in vitreous mass of VEGF between mCNV group and control group was found on the basis that the vitreous concentration was replaced by aqueous humor concentration when estimated the vitreous mass. However, in eyes with longer axial length (mCNV group), the concentration in the vitreous was higher than that in aqueous humor due to the dilution effect, thus the actual vitreous mass should be higher than the estimated mass. And in eyes with normal axial length (control group), the actual mass should be close to the estimated mass due to the similar quantities of major proteins presented in both aqueous and vitreous fluid. Therefore, a more significant difference in the actual vitreous mass of VEGF should be presented between the two groups, remaining consistent with the results of this study. Secondly, for patients with posterior scleral staphyloma, there may be an error in the measurement of the axial length. Zhou et al. included and scanned 290 eyes with and without posterior pole staphyloma by high-resolution 3D-MRI in the establishment of the model. The formula applied to the majority of HM and our study was to further estimate the mass on this basis. The results should be more accurate if each patient underwent 3D-MRI to obtain vitreous volumes, however we have reason to believe that there should be not much deviation. Thirdly, this is a cross-sectional study, and thus no causal inferences could be made. Finally, we examined only six cytokines, but t but other cytokines might have a stronger impact on the development of HM and mCNV.

In conclusion, we confirmed the increase of VEGF secretion in mCNV eyes from the perspective of vitreous mass which might be tried as a more reliable potential biomarker in eyes with longer axial length. Different inflammatory cytokines were elevated in different stages of high myopias. IL-8 and IP-10 were upregulated earlier than the upregulation of VEGF in high myopia, suggesting that the progression of high myopia and the occurrence and development of CNV may be related to an early low-grade inflammation, which provided potential clinical prevention and treatment of mCNV.

## Data availability statement

The raw data supporting the conclusions of this article will be made available by the authors, without undue reservation.

## Ethics statement

The studies involving human participants were reviewed and approved by Zhejiang Provincial People's Hospital Ethics Committee. The patients/participants provided their written informed consent to participate in this study.

## Author contributions

SZ, JM, and LS designed the study, performed manuscript review, and revision. SZ, YF, YC, and ZZ performed data collection, data analysis, and interpretation. SZ, NC, and YC performed the study. All authors read and approved the final manuscript.

## Funding

This work was supported by the Medical and Health Platform Project of Zhejiang Province (2021KY810).

## Conflict of interest

The authors declare that the research was conducted in the absence of any commercial or financial relationships that could be construed as a potential conflict of interest.

## Publisher's note

All claims expressed in this article are solely those of the authors and do not necessarily represent those of their affiliated organizations, or those of the publisher, the editors and the reviewers. Any product that may be evaluated in this article, or claim that may be made by its manufacturer, is not guaranteed or endorsed by the publisher.

## References

[1] Wu PC Huang HM Yu HJ Fang PC Chen CT. Epidemiology of Myopia. Asia Pac J Ophthalmol (Phila). (2016) 5:386–93. 10.1097/APO.0000000000000236 27898441

[2] Huang W Duan A Qi Y. Posterior Scleral Reinforcement to Prevent Progression of High Myopia. Asia Pac J Ophthalmol (Phila). (2019) 8:366–70. 10.1097/APO.0000000000000257 31513040 PMC6784774

[3] Ohno-Matsui K Ikuno Y Lai TYY Gemmy Cheung CM. Diagnosis and treatment guideline for myopic choroidal neovascularization due to pathologic myopia. Prog Retin Eye Res. (2018) 63:92–106. 10.1016/j.preteyeres.2017.10.005 29111299

[4] Wei Q Jiang C Ye X Huang X Jin H Xu G. Vitreous proteomics provides new insights into antivascular endothelial growth factor therapy for pathologic myopia choroid neovascularization. J Interferon Cytokine Res. (2019) 39:786–96. 10.1089/jir.2019.0030 31718389

[5] Ikuno Y. Overview of the complications of high. Myopia. Retina. (2017) 37:2347–51. 10.1097/IAE.0000000000001489 28590964

[6] Mao J Zhang S Zheng Z Deng X Liu C Chen Y. Prediction of anti-VEGF efficacy in diabetic macular oedema using intraocular cytokines and macular optical coherence tomography. Acta Ophthalmol. (2021). 10.1111/aos.15008 34403203

[7] Udaondo P Hernández C Briansó-Llort L García-Delpech S Simó-Servat O Simó R. Usefulness of liquid biopsy biomarkers from aqueous humor in predicting anti-VEGF response in diabetic macular edema: results of a pilot study. J Clin Med. (2019) 8:1841. 10.3390/jcm8111841 31684007 PMC6912573

[8] Zhu X Du Y Truscott RJW He W Zhou P Lu Y. Profiling and bioinformatic analysis of differentially expressed cytokines in aqueous humor of high myopic eyes - clues for anti-VEGF injections. Curr Eye Res. (2020) 45:97–103. 10.1080/02713683.2019.1648833 31405302

[9] Sawada O Kawamura H Kakinoki M Sawada T Ohji M. Vascular endothelial growth factor in the aqueous humour in eyes with myopic choroidal neovascularization. Acta Ophthalmologica. (2011) 89:459–62. 10.1111/j.1755-3768.2009.01717.x 20102348

[10] Yuan J Wu S Wang Y Pan S Wang P Cheng L. Inflammatory cytokines in highly myopic eyes. Scientific reports. (2019) 9:3517. 10.1038/s41598-019-39652-x 30837544 PMC6400944

[11] Liu C Zhang S Deng X Chen Y Shen L Hu L. Comparison of intraocular cytokine levels of choroidal neovascularization secondary to different retinopathies. Front Med. (2021) 8:783178. 10.3389/fmed.2021.783178 34993212 PMC8725795

[12] Agrawal R Balne PK Wei X Bijin VA Lee B Ghosh A. Cytokine profiling in patients with exudative age-related macular degeneration and polypoidal choroidal vasculopathy. Invest Ophthalmol Vis Sci. (2019) 60:376–82. 10.1167/iovs.18-24387 30682207

[13] Ando Y Keino H Inoue M Hirota K Takahashi H Sano K. Circulating vitreous microRNA as possible biomarker in high myopic eyes with macular hole. Int J Mol Sci. (2022) 23:14. 10.3390/ijms23073647 35409006 PMC8998168

[14] Zhou J Tu Y Chen Q Wei W. Quantitative analysis with volume rendering of pathological myopic eyes by high-resolution three-dimensional magnetic resonance imaging. Medicine. (2020) 99:e22685. 10.1097/MD.0000000000022685 33080714 PMC7572025

[15] Pollreisz A Funk M Breitwieser FP Parapatics K Sacu S Georgopoulos M. Quantitative proteomics of aqueous and vitreous fluid from patients with idiopathic epiretinal membranes. Exp Eye Res. (2013) 108:48–58. 10.1016/j.exer.2012.11.010 23201028

[16] Wu F Phone A Lamy R Ma D Laotaweerungsawat S Chen Y. Correlation of aqueous, vitreous, and plasma cytokine levels in patients with proliferative diabetic retinopathy. Invest Ophthalmol Vis Sci. (2020) 61:26. 10.1167/iovs.61.2.26 32084272 PMC7326572

[17] Neelam K Cheung CM Ohno-Matsui K Lai TY Wong TY. Choroidal neovascularization in pathological myopia. Prog Retin Eye Res. (2012) 31:495–525. 10.1016/j.preteyeres.2012.04.001 22569156

[18] Yamamoto Y Miyazaki D Sasaki S Miyake K Kaneda S Ikeda Y. Associations of inflammatory cytokines with choroidal neovascularization in highly myopic eyes. RETINA. (2015) 35:344–50. 10.1097/iae.0000000000000311 25289657

[19] Wakabayashi T Ikuno Y Oshima Y Hamasaki T Nishida K. Aqueous concentrations of vascular endothelial growth factor in eyes with high myopia with and without choroidal neovascularization. J Ophthalmol. (2013) 2013:257381. 10.1155/2013/257381 23533702 PMC3606763

[20] Tong JP Chan WM Liu DT Lai TY Choy KW Pang CP. Aqueous humor levels of vascular endothelial growth factor and pigment epithelium-derived factor in polypoidal choroidal vasculopathy and choroidal neovascularization. Am J Ophthalmol. (2006) 141:456–62. 10.1016/j.ajo.2005.10.012 16490490

[21] Zhu D Yang DY Guo YY Zheng YF Li JL Wang B. Intracameral interleukin 1beta, 6, 8, 10, 12p, tumor necrosis factor alpha and vascular endothelial growth factor and axial length in patients with cataract. PLoS ONE. (2015) 10:e0117777. 10.1371/journal.pone.0117777 25679504 PMC4332662

[22] Wei Q Zhuang X Fan J Jiang R Chang Q Xu G. Proinflammatory and angiogenesis-related cytokines in vitreous samples of highly myopic patients. Cytokine. (2021) 137:155308. 10.1016/j.cyto.2020.155308 33128924

[23] Obadă O Pantalon AD Rusu-Zota G Hăisan A Lupuşoru SI Constantinescu D. Aqueous humor cytokines in non-proliferative diabetic retinopathy. Medicina. (2022) 58:909. 10.3390/medicina58070909 35888628 PMC9324281

[24] Zhao F Wu H Reinach PS Wu Y Zhai Y Lei Y. Up-Regulation of matrix metalloproteinase-2 by scleral monocyte derived macrophages contributes to myopia development. Am J Pathol. (2020) 190:1888–908. 10.1016/j.ajpath.2020.06.002 32553806

[25] Li Y Ren X Zhang Z Duan Y Li H Chen S. Effect of small extracellular vesicles derived from IL-10-overexpressing mesenchymal stem cells on experimental autoimmune uveitis. Stem Cell Res Ther. (2022) 13:100. 10.1186/s13287-022-02780-9 35255957 PMC8900327

[26] Shchuko AG Zaitseva NV Yurieva TN Chernykh ER Mikhalevich IM Shevela EY. Intraocular cytokines and their correlations with clinical parameters in patients with myopic choroidal neovascularization. Ophthalmologica. (2017) 237:96–104. 10.1159/000455271 28103602

